# A critical assessment of nanoparticles enhanced phase change materials (NePCMs) for latent heat energy storage applications

**DOI:** 10.1038/s41598-023-34907-0

**Published:** 2023-05-15

**Authors:** Muritala Alade Amidu, Mohamed Ali, Ahmed K. Alkaabi, Yacine Addad

**Affiliations:** 1 Department of Nuclear Engineering, Khalifa University of Science and Technology, Abu Dhabi, United Arab Emirates; 2 Emirates Nuclear Technology Center (ENTC), Khalifa University of Science and Technology, Abu Dhabi, United Arab Emirates

**Keywords:** Energy science and technology, Engineering, Nanoscience and technology

## Abstract

Phase change material (PCM) laden with nanoparticles has been testified as a notable contender to increase the effectiveness of latent heat thermal energy storage (TES) units during charging and discharging modes. In this study, a numerical model is developed and implemented based on the coupling between an advanced two-phase model for the nanoparticles-enhanced PCM (NePCM) and the enthalpy-porosity formulation for the transient behavior of the phase change. Therefore, a porosity source term is added to the nanoparticles transport equation to account for the particles' frozen state in regions occupied by solid PCM. This two-phase model includes three main nanoparticles’ slip mechanisms: Brownian diffusion, thermophoresis diffusion, and sedimentation. A two-dimensional model of a triplex tube heat exchanger is considered and different charging and discharging configurations are analyzed. Compared to pure PCM, results show a substantial heat transfer enhancement during the charging and discharging cycle in which a homogeneous distribution of nanoparticles is considered as the initial condition. For this case, the two-phase model predictions are superior to the ones obtained with the classical single-phase model. In the case of multi-cycle charging and discharging, a significant deterioration of the heat transfer rate is observed using the two-phase model while such assessment is senseless using the single-phase mixture model due to the physical assumptions upon which this model is formulated. The two-phase model results reveal that, for a NePCM with high nanoparticles concentration (> 1%), the melting performance during the second charging cycle is reduced by 50% compared to the first one. This performance degradation is attributed to a noteworthy non-homogeneous distribution of the nanoparticles at the beginning of the second charging cycle. The dominant nanoparticles migration mechanism, in this scenario, is the one resulting from sedimentation effects.

## Introduction

The alarming recent climate changes and environmental issues are the main drivers for the ongoing research race to develop; clean, sustainable, and more efficient energy systems. Consequently, the current rightly-justified tendency is to reduce the use of fossil energies and progress towards full integration of renewable and sustainable energy sources for electric power production as well as for other industrial applications. However, these energy resources are admittingly recognized to face some dreadful drawbacks in terms of intermittency (sun and wind availability) for renewable energy sources and a variable load-following capability for the nuclear-based sustainable energy source. Hence, latent heat thermal energy storage (TES) is getting increasing attention as a promising solution to overcome these shortcomings and advance toward an economically competitive hybrid-energy system.

Latent heat TES components, which make use of phase change materials (PCMs) to store or release energy, are more attractive due to their high energy storage capacity, wide operating temperature range, and recycling possibility^1^. Therefore, PCM-based TES systems are currently being considered for various applications such as building cooling/heating^2^, thermal control of electronic devices^3^, solar thermal storage^4^, and load-following capability enhancement for nuclear power plants^5–9^.

Nevertheless, a major downside of today’s used PCMs is the corresponding low thermal conductivity values which limit their use for energy storage applications. This hindrance causes a reduction in the melting/solidification rates which is then mirrored in the TES response time getting too long. To address this issue, two main strategies are proposed in the literature. The first one employs enhanced coolant in an attempt to speed up the charging/discharging processes^10^. An example of such an approach was reported in the work of Addad et al.^11^ in which the authors investigated the effects of using a nanofluid coolant on the performance of a packed-bed energy storage container. Thus, an in-house code, which couples the single-phase model for the nanofluid with the enthalpy-porosity method for the PCM, was developed. By using the nanofluid as a coolant, the charging and discharging cycle was found to require a shorter time. They reported that the amount of enhancement depends on the nanoparticles concentration because the time needed for the charging-discharging cycle was reduced by 10% and 20% for nanoparticle concentrations of 3% and 5%, respectively.

The second approach to overcome these PCM-associated slow-melting/solidification rates is by either altering the PCM thermophysical properties or by performing geometrical modifications to the PCM containers (increasing the total contact surface). For the latter, many performance enhancements have been reported in the literature^12^ by applying design optimization techniques to the PCM containers. For instance, internal and external fins with different geometries and shapes were widely studied^13^. Also, different geometries such as rectangular/trapezoidal^14^, spherical^15^, eccentric tubes^16^, and nonregular tube shape^17,18^ were investigated to obtain the most suitable geometrical configuration for the PCM containers. Alternatively, an option entailing the addition of nanoparticles to the base phase-changing materials was also pursued. This option is expected to positively alter the resulting NePCM’s (mixture of nanoparticles and PCM) thermophysical properties (principally its thermal conductivity). Tariq and co-authors conducted a comprehensive literature review on the preparation techniques of nanoparticles enhanced phase change materials (NePCMs) along with their applications in various fields^19^. Hereafter, a concise review, describing the state-of-the-art research activities on NePCMs for TES systems, is provided.

The nanofluid concept was first introduced back in 1995^20^. It consists of mixing solid nanoparticles with a carrier base fluid^20,21^. The main advantage of this mixture (nanoparticles doped in PCM) is the resulting improvement in its thermophysical properties such as thermal conductivity and viscosity^22–26^. In their original work, Khanafer et al.^27^ developed a single-phase model to numerically study heat transfer enhancement in a two-dimensional enclosure using nanofluids. Although several important mechanisms such as agglomeration, sedimentation, and so forth, were not accounted for in their physical model, Khanafer et al. were essentially able to highlight the enhancement capacity of nanofluids and their promising utility. Since then, the usage of nanofluid has been investigated in many applications to achieve similar heat transfer enhancement rates. A non-exhaustive list of examples includes, amongst others, solar collectors^28–30^, heat exchangers^31^, and electronic component cooling^32,33^.

The use of nanoparticles as an enhancement technique for the PCMs was firstly investigated by Khodadadi and Hosseinizadeh^34^. They numerically investigated the effects of incorporating copper nanoparticles into the water during its freezing process. The single-phase model, which assumes a homogeneous and isotropic distribution of the nanoparticles within the carrier fluid, was used in their study. The reported predictions revealed that a higher heat release rate and higher thermal conductivity of the NePCM were achieved in comparison with pure water. In the experimental study of the behavior of paraffin as PCM doped with copper nanoparticles^35^, a significant reduction of melting and solidification time (by about 32%) was achieved when 1% of copper nanoparticles was added to the base PCM. The thermal conductivity was also improved in the liquid and solid phases by 18% and 14%, respectively.

Also, the effects of incorporating alumina nanoparticles on the solidification of PCM in triplex tube TES have been previously studied^36^. The results of this study showed that using nanoparticles of volumetric concentration ranging from 3 to 8% resulted in a time-saving of 8–20% for solidification. The effect of the nanoparticles was small at the early stages of solidification but increased as the process advanced in time. A similar heat transfer enhancement was reported during the melting process^37^. In these previous investigations, the theoretical model of the mixture (i.e., single-phase model) with prescribed formulations for the density, latent heat, specific heat capacity, and thermal expansion coefficient of the NePCM, was used. As for the thermal conductivity and dynamic viscosity, the empirical correlations, put forward by Vajjha et al.^38,39^, were implemented. Accordingly, the thermal conductivity correlation selected in these studies does take into account the effects of the nanoparticles’ Brownian motion, size, and volume fraction.

In addition, a numerical study to examine the effects of dispersing nanoparticles on the performance of a latent heat storage system^40^ has also been reported. It was found that adding 5% of Cu, CuO, and Al_2_O_3_ nanoparticles enhanced significantly the melting and solidification rates compared to pure paraffin. The melting time was 10 times, 3.5 times, and 2.25 times faster with Cu, CuO, and Al_2_O_3_ nanoparticles, respectively. Accordingly, the solidification process was reduced by 8 times, 3 times, and 1.7 times, respectively compared to the latent heat storage system with pure paraffin. Also, in that investigation, the authors used the single-phase model in which the agglomeration and sedimentation migration processes are neglected. Hence, the properties of the nanofluid were assumed homogeneous and isotropic as these are the two main assumptions upon which the single-phase model is predicated. Likewise, in another investigation of the enhancement of the PCM melting using graphene nanoparticles dispersed into paraffin wax^14^, it was found that the heat transfer performance was significantly improved for nanofluid thermal conductivity enhancement by more than 80%.

The nano-enhancement of the solidification process of a PCM-based heat exchanger has also been studied^41^ using Cu nanoparticles. Also in this study, the single-phase model was used. Effective thermal conductivity and dynamic viscosity were given by empirical correlations. The results showed a decrease in the solidification time by 8% and 15% using nanoparticles concentrations of 2% and 4%, respectively. This enhancement was related to the improvement of the NePCM conductivity and the decrease of this mixture's latent heat capacity.

Moreover, strategies combining both geometrical design improvement for the containers and the use of NePCMs, have also been reported in the literature^42^ in which the effect of fins and nanoparticles on the charging process of PCM contained in a triplex tube was investigated. A numerical model based on a single-phase approach with empirical correlation for viscosity and thermal conductivity was used. It was reported that the thermal conductivity of the PCM increased by 25% when loaded with 10% of nanoparticles alumina (Al_2_O_3_). Furthermore, it was found that the melting time, for the container geometry with external fins, was further reduced by up to 17% due to the addition of nanoparticles. Furthermore, the investigation of the effect of using hybrid nanoparticles (MoS_2_–Fe_3_O_4_) in addition to internal fins, on the solidification process of water has also been reported in the literature^18^. A Galerkin finite element method was used to solve the governing equation and a single-phase model was assumed with different hybrid nanoparticle concentrations. An improvement of 4% in the solidification rate was found using a 5% nanoparticles concentration. Also, Kheshteli et al.^17^ investigated the solidification rate enhancement using nanoparticles and wavy triplex tubes. The numerical predictions showed that the geometric changes in the tubes using wavy shapes can result in lower solidification time. A combination of wavy tubes with nanoparticles resulted in a better heat transfer rate. Thus, increasing the nanoparticles' volume fraction from 0 to 5% reduced the solidification time by 20%. Again, the single-phase, with mixture relations for the density, specific heat, and latent heat was used. As for the thermal conductivity and dynamic viscosity, the authors used the same empirical correlations as the ones chosen by Khodadadi et al.^34^.

Harikrishnan et al.^43^ studied the effect of the multicycle processes on the thermal energy storage behavior of the stearic acid-TiO_2_ nanofluid. They reported that the maximum change of melting temperature and solidification temperature, during 5000 cycles, was − 0.35 and − 0.59%, respectively. Also, the maximum change of the latent heat during the 5000 cycles was 1.29 and 1.47% for melting and solidification, respectively. Improved performance of the nano-enhanced PCM was observed: the melting time was reduced by 43.72% and the solidification time was reduced by 41.39% for the 0.3% nanoparticles concentration. Similar findings were reported by Harikrishnan et al.^44^ for the myristic acid-SiO_2_ nanofluid.

Hence, from the aforementioned previous studies, one can conclude that NePCMs offer an attractive solution to overcome the slow time response of the TES during the charging/discharging modes. Furthermore, if geometrically optimized containers are filled with these NePCMs, the resulting enhancement would be even greater. On the other hand, this claimed enhancement achieved by using NePCMs is only qualitative. This stems from the fact that all the numerical studies carried out to date are based on the classical single-phase physical model in which the different slip mechanisms such as Brownian diffusion, thermophoresis diffusion, and sedimentation of nanoparticles are not accounted for. In fact, this model (single-phase model) has been demonstrated^45–47^ to lack the ability to predict the correct magnitude of the heat transfer enhancement, particularly, for nanoparticle concentrations higher than 1%. An even more serious concern is the fact that the single-phase model is not capable of predicting the correct NePCMs behavior during multi-charging/discharging cycles. Therefore, falling short of an accurate representation of the expected physical phenomena in NePCM-based TES when operated over a relatively long period. That is because a homogeneous nanoparticles distribution is always assumed, hence, the outcome from a multi-cycle simulation would be meaningless as the predictions of all consecutive cycles will be simply identical to each other. On the other hand, a more advanced two-phase model taking into account the Brownian diffusion and thermophoresis diffusion slip mechanisms as originally proposed by Buongiorno^45^, then further improved for numerical stability by Riahi et al.^48^, and upgraded by Amidu et al.^47^ to also include nanoparticles sedimentation effects, would be more suitable for such multi-cycle simulations. Therefore, in this paper, the advanced two-phase model of Amidu et al.^47^, considering the three aforementioned slip mechanisms of nanoparticles, is combined with an enthalpy-porosity method to better understand the effect of different mechanisms on the NePCM performance. To justify the assertions mentioned above, a comparison between the two nanofluid physical models, namely; the single-phase and the two-phase one is also carried out. Finally, the effect of the nanoparticles on the NePCM performance during a multi-cycle scenario is investigated.

## Model description

The application of the NePCM would involve several cycles of charging and discharging processes over a long period. Thus, one must take into consideration the potential migration of the nanoparticles within the PCM by, sedimentation, Brownian diffusion, and thermophoresis diffusion. This is because the occurrence of these nanoparticle transport phenomena causes non-homogeneity in the distribution of nanoparticles within the PCM. In fact, this renders the single-phase model’s assumptions inconsequential. Therefore, in this study, the two-phase model proposed by Amidu et al.^47^ is adapted for the prediction of the thermal features of the NePCM. Nevertheless, for evaluation purposes, numerical predictions using the single-phase model, are also presented in this paper.

A summary of the governing equations of the two single- and two-phase models, is provided in Table 1. A detailed description of these models has been reported in the study by Amidu et al.^47^ and is, therefore, not repeated here. However, the main distinguishing features of these two models are that the single-phase model assumes a thermal equilibrium between nanoparticles and the base (PCM). It also assumes that these nano-scale particles are uniformly distributed within the PCM without any slip motion. Thus, then PCM laden with nanoparticles is considered a single fluid with thermophysical properties equivalent to that of the nanoparticles-PCM mixture as shown in Table 2.

**Table 1:** Governing equations of the models^47,49^.

Governing equations	Single-phase model	Two-phase model
Continuity	$\nabla \cdot u=0$	$\frac{\partial {\rho }_{m}}{\partial t}+\nabla \cdot \left({\rho }_{m}{u}_{m}\right)=0$
Momentum	$\frac{\partial u}{\partial t}+\nabla \cdot \left(uu\right)=-\nabla \left(\frac{P}{{\rho }_{m}}\right)+\nabla \cdot \left(\frac{{\mu }_{m}}{{\rho }_{m}}\nabla u\right)+{g}_{k}+{S}_{m}$	$$\frac{\partial \left({\rho }_{m}{u}_{m}\right)}{\partial t}+\nabla \cdot \left({\rho }_{m}{u}_{m}{u}_{m}\right)=-\nabla P+\nabla \cdot \left({\mu }_{m}\nabla {u}_{m}\right)+\nabla \cdot \left(\begin{array}{c}\varphi {\rho }_{p}{u}_{pm}{u}_{pm}+\\ \varphi {\rho }_{p}{u}_{T}{u}_{T}+\\ \varphi {\rho }_{p}{u}_{B}{u}_{B}\\ \left(1-\varphi \right){\rho }_{bf}{u}_{bfm}{u}_{bfm}\end{array}\right)+{\rho }_{m}{g}_{k}+{S}_{m}$$ ${u}_{pm}=\frac{{\rho }_{bf}}{{\rho }_{m}}{u}_{s}{10}^{-A\varphi }$, ${u}_{bfm}=\frac{\varphi {\rho }_{p}}{\left(1-\varphi \right){\rho }_{f}}{u}_{pm}$, ${u}_{s}=\frac{{d}_{p}^{2}\left({\rho }_{p}-{\rho }_{bf}\right)}{18{\mu }_{bf}}g$, ${u}_{B}=-{D}_{B}\nabla \varphi$,${u}_{T}=-{S}_{T}\frac{{\mu }_{m}}{{\rho }_{m}}\frac{\nabla {T}_{m}}{{T}_{m}}$
Energy	$\frac{\partial T}{\partial t}+\nabla \cdot \left(Tu\right)=\nabla \cdot \left(\frac{{k}_{m}}{{\rho }_{m}{C}_{pm}}\nabla T\right)+{S}_{h}-\nabla \cdot {q}_{t}$	$\frac{\partial \left({\rho }_{m}{C}_{pm}{T}_{m}\right)}{\partial t}+\nabla \cdot \left({\rho }_{m}{u}_{m}{C}_{pm}{T}_{m}\right)=\nabla \cdot \left({k}_{m}\nabla T\right)-{C}_{pp}{J}_{p}\cdot \nabla {T}_{m}+{S}_{h}-\nabla \cdot {q}_{t}$ ${J}_{p}={\rho }_{p}\left({D}_{B}\nabla \varphi +{D}_{T}\frac{\nabla {T}_{m}}{{T}_{m}}\right)={\rho }_{p}\left({u}_{B}+\varphi {u}_{T}\right)$, ${D}_{B}=\frac{{k}_{B}{T}_{m}}{3\pi {\mu }_{bf}{d}_{p}}$, ${D}_{T}=\beta \frac{{\mu }_{m}}{{\rho }_{m}}\varphi$
Nanoparticle transport		$\frac{\partial \left({\rho }_{p}\varphi \right)}{\partial t}+\nabla \cdot \left(\varphi {\rho }_{p}{u}_{m}\right)=-\nabla \cdot \left(\varphi {\rho }_{p}{u}_{pm}+\varphi {\rho }_{p}{u}_{T}+\varphi {\rho }_{p}{u}_{B}\right)+{S}_{pm}$ with ${S}_{pm}=-\left({u}_{m}+{u}_{pm}+{u}_{T}+{u}_{B}\right)C\frac{{\left(1-\alpha \right)}^{2}}{{\alpha }^{3}+b}$
Liquid fraction	$\alpha =0.5\cdot \mathrm{erf}\left(\frac{4\left(T-{T}_{me}\right)}{{T}_{l}-{T}_{s}}\right)+0.5$	$\alpha =0.5\cdot \mathrm{erf}\left(\frac{4\left({T}_{m}-{T}_{me}\right)}{{T}_{l}-{T}_{s}}\right)+0.5$
Closure Parameters		
Buoyancy term	${g}_{k}=\left[1-{\beta }_{m}\left(T-{T}_{ref}\right)\right]g$	${g}_{k}=\left[1-{\beta }_{m}\left({T}_{m}-{T}_{ref}\right)\right]g.$
Enthalpy source term	${S}_{h}=-\rho L\frac{4.\mathrm{exp}\left({\left(\frac{4\left(T-{T}_{me}\right)}{{T}_{l}-{T}_{s}}\right)}^{2}\right)}{\left({T}_{l}-{T}_{s}\right)\sqrt{\pi }}\cdot \left(\frac{\partial T}{\partial t}+u\nabla T\right)$	${S}_{h}=-\rho L\frac{4.\mathrm{exp}\left({\left(\frac{4\left({T}_{m}-{T}_{me}\right)}{{T}_{l}-{T}_{s}}\right)}^{2}\right)}{\left({T}_{l}-{T}_{s}\right)\sqrt{\pi }}\cdot \left(\frac{\partial {T}_{m}}{\partial t}+{u}_{m}\nabla {T}_{m}\right)$,
Momentum source term	${S}_{m}=-uC\frac{{\left(1-\alpha \right)}^{2}}{{\alpha }^{3}+b}$	${S}_{m}=-{u}_{m}C\frac{{\left(1-\alpha \right)}^{2}}{{\alpha }^{3}+b}$

**Table 2:** Correlations for mixture physical properties^42^.

Properties	Correlations
Dynamic viscosity	$\frac{{\mu }_{m}}{{\mu }_{bf}}=0.983{e}^{12.959\varphi }$
Thermal conductivity	$\frac{{k}_{m}}{{k}_{bf}}=\frac{{k}_{np}+2{k}_{bf}-2\left({k}_{bf}-{k}_{np}\right)\varphi }{{k}_{np}+2{k}_{bf}+2\left({k}_{bf}-{k}_{np}\right)\varphi }+{B}_{r}$ ${B}_{r}=3\times {10}^{3}\alpha \varphi {\rho }_{bf}{C}_{pbf}\sqrt{\frac{{k}_{B}T}{{\rho }_{np}{d}_{p}}}f(T,\varphi )$ ${k}_{B}=1.381\times {10}^{-23} \mathrm{J}/\mathrm{K}$ $f\left(T,\varphi \right)=\left(0.028217\varphi +0.00391\right)\frac{T}{{T}_{Ref}}+\left(-0.030669\varphi -0.00391123\right)$
Volumetric expansion coefficient	${{\beta }_{m}=(\left(1-\varphi \right){\rho }_{bf}{\beta }_{bf}+\varphi {\rho }_{p}{\beta }_{p})/}_{{\rho }_{m}}$
Density	${\rho }_{m}=\left(1-\varphi \right){\rho }_{bf}+\varphi {\rho }_{p}$
Specific heat capacity	${C}_{pm}=(\left(1-\varphi \right){\rho }_{bf}{C}_{pbf}+\varphi {{\rho }_{p}C}_{pp})/{\rho }_{m}$

In contrast to the single-phase model, the potential slip motions of nanoparticles due to sedimentation, Brownian diffusion, and thermophoresis are considered and modeled in a two-phase approach. The contributions of these nanoparticles transport phenomena are well reflected in the two-phase model governing equations in terms of the nanoparticle slip velocities ${u}_{pm}$, ${u}_{B}$ and ${u}_{T}$ corresponding to sedimentation, Brownian diffusion, and thermophoretic diffusions, respectively. This way the local concentrations of the nanoparticles determine their local thermophysical properties using the mixture expression in Table 2. Note that for thermal conductivity, $Br=0$ when the two-phase model is used. Both modeling approaches are implemented in the solution framework of OpenFOAM CFD code version 6.

### Modeling of melting/solidification of the PCM and validation

In the single-phase and two-phase models, the enthalpy-porosity modeling approach is used to capture the melting and solidification processes of the PCM under isothermal boundary conditions. Two key parameters in the modeling of phase transition in PCM are the porosity source term (${S}_{m}$) in the momentum equation and enthalpy source term (${S}_{h}$) in the energy equation (see Table 1). Note that the porosity has no physical interpretation as it is merely used in the context of the momentum equation as a numerical expression to enforce a zero velocity in the solid region. Thus, any mathematical expression with similar characteristics can be used in this case. To ensure a smooth transition from the solid phase ($\alpha =0$) to the liquid phase ($\alpha =1$) and vice versa, an error function of the liquid fraction which was proposed in the previous studies^49,50^ is also adopted here. This error function is a function of the liquidus temperature (${T}_{l}$), solidus temperature (${T}_{s}$) and the melting temperature (${T}_{m}$) of the PCM. With the error function, the flow is governed by the Carman–Kozeny equation (a form of the Darcy equation) when the liquid fraction lies between liquid and solid states ($0<\alpha <1$). In the porosity source term, the constants $C$ and $b$ have recommended values of $1.6\times {10}^{6}$ and 0.001, respectively. The numerical constant $b$ is used in this case to avoid division by zero error.

Firstly, the melting/solidification model is validated against experimental data of melting pure Gallium in a rectangular cavity^51^. A 2D simulation of the experimental rectangular geometry (X = 88.9 mm and Y = 44.45 mm) is performed using the single-phase model since this experiment did not use nanoparticles. Isothermal boundary conditions are imposed on the right and left walls as ${T}_{hot}=311.15 K$ and ${T}_{cold}=301.15 K$, respectively while the top and bottom walls are justifiably assumed to be adiabatic since they are insulated in the experiment. The solid Gallium is at an initial temperature of 301.45 K prior to the start of the simulation. After the mesh sensitivity study, an optimum mesh resolution of 210 × 110 is used for the simulation with an adaptive time step scheme. The thermophysical properties of the pure Gallium used for this simulation are shown in Table 3.

**Table 3:** Thermophysical properties of pure Gallium^51^.

Properties	Values
Specific heat capacity, ${C}_{p}$, J kg^−1^ K^−1^	381.5
Thermal conductivity, $k$, W mK^−1^	28.0
Density, $\rho$, kg m^−3^	6093
Dynamic viscosity, $\mu$, kg ms^−1^	2.97 × 10^–7^
Latent heat, L, J kg^−1^ K^−1^	80,160
Volumetric expansion coefficient, $\beta$, K^−1^	1. 2 × 10^–4^
Solidus, ${T}_{s}$, K	302.88
Liquidus, ${T}_{l}$, K	302.98

**Figure 1:**
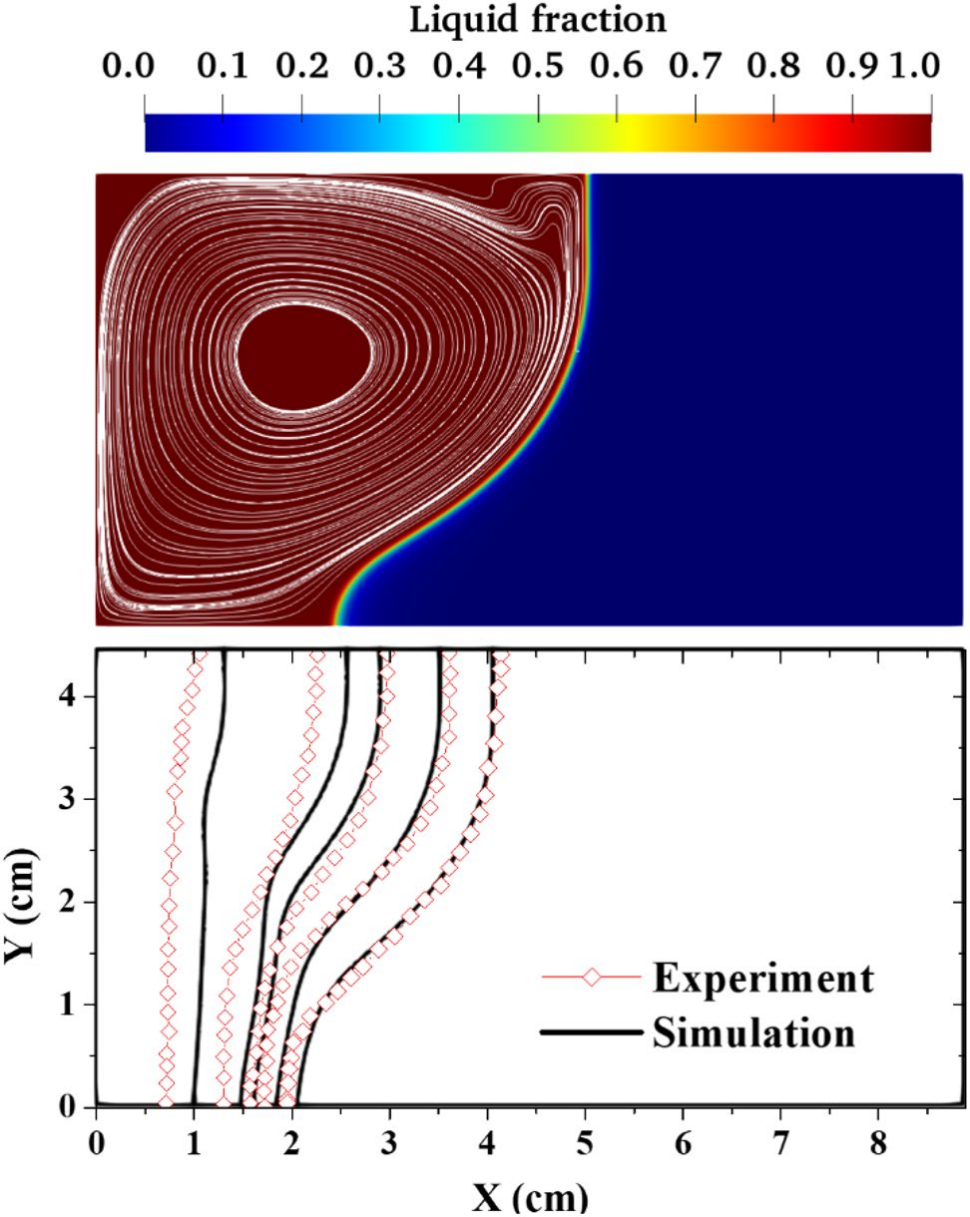
Validation of the melt front prediction showing the Contour of the liquid fraction showing the melt front (top) and comparison of the predicted melt fronts with experimental data (bottom).

The contour of the liquid fraction of the Gallium after 10 min of simulation is shown in Fig. 1 (top) which indicates that the melting of Gallium proceeds faster in the upper part of the cavity due to the penetrative effect of the natural convection of the melted Gallium which is driven by the buoyancy force. In addition, the comparison of the predicted transient melt front with experimental measurements shows fair agreement as can be seen in Fig. 1 (bottom). Thus, it can be safely asserted that the enthalpy-porosity model (enhanced with the error function of the liquid fraction) can capture the melting and solidification process in any PCM.

### Modeling of nanoparticle transport phenomena and validation

As stated above, sedimentation, Brownian diffusion, and thermophoresis play a significant role in the migration of nanoparticles within the base fluid. The sedimentation causes nanoparticles to settle at the bottom of the container and this is driven by the gravitation force acting on the nanoparticles. In the two-phase model, the relative velocities (see Table 1) of the nanoparticle and PCM resulting from the settling of the nanoparticles are predicted using Vesilind’s sedimentation model^52^. Two key parameters of the Vesilind sedimentation model are the terminal settling velocity vector and the settling coefficient which are represented by ${u}_{s}$ and $A$, respectively. The terminal settling velocity (${u}_{s}$) is determined by the balance of buoyancy and viscous force while the settling coefficient ($A=100$) is determined from the expression proposed in our previous study^47^. The sedimentation of nanoparticles tends to cause non-uniformity in the nanoparticle’s distribution within the base fluid.

In addition, the nanoparticles are also presumed to undergo Brownian diffusion which causes nanoparticles to migrate from regions of higher concentration to regions of lower concentration. Thus, the driving force of this transport phenomenon is the nanoparticles' local concentration. This way, the Brownian diffusion tends to make the distribution of nanoparticles uniform and if strong enough, counterbalance other factors causing the non-uniformity. The slip velocity of the nanoparticles due to the Brownian diffusion is determined by ${u}_{B}=-{D}_{B}\nabla \varphi$ where the Brownian diffusion coefficient is defined in terms of the nanoparticle’s diameter as ${D}_{B}=\frac{{k}_{B}{T}_{m}}{3\pi {\mu }_{bf}{d}_{p}}$. More so, due to the temperature gradient that might exist in the system, nanoparticles can also move from the regions of lower temperature gradients to the region of higher temperature gradients. This transport phenomenon is known as thermophoresis. Thus, the driving force of this mechanism is the nano PCM temperature gradient. The relative velocity of the nanoparticle due to thermophoresis is determined by ${u}_{T}=-{S}_{T}\frac{{\mu }_{m}}{{\rho }_{m}}\frac{\nabla {T}_{m}}{{T}_{m}}$. One key parameter of thermophoresis is the thermophoretic coefficient (${S}_{T}=0.08$) which is calculated using the expression proposed in our previous study^47^. Succinctly, these nanoparticles’ relative velocities due to sedimentation, Brownian diffusion, and thermophoresis are incorporated into the governing equations of the drift-flux model implemented in OpenFOAM CFD as shown in Table 1.

**Figure 2:**
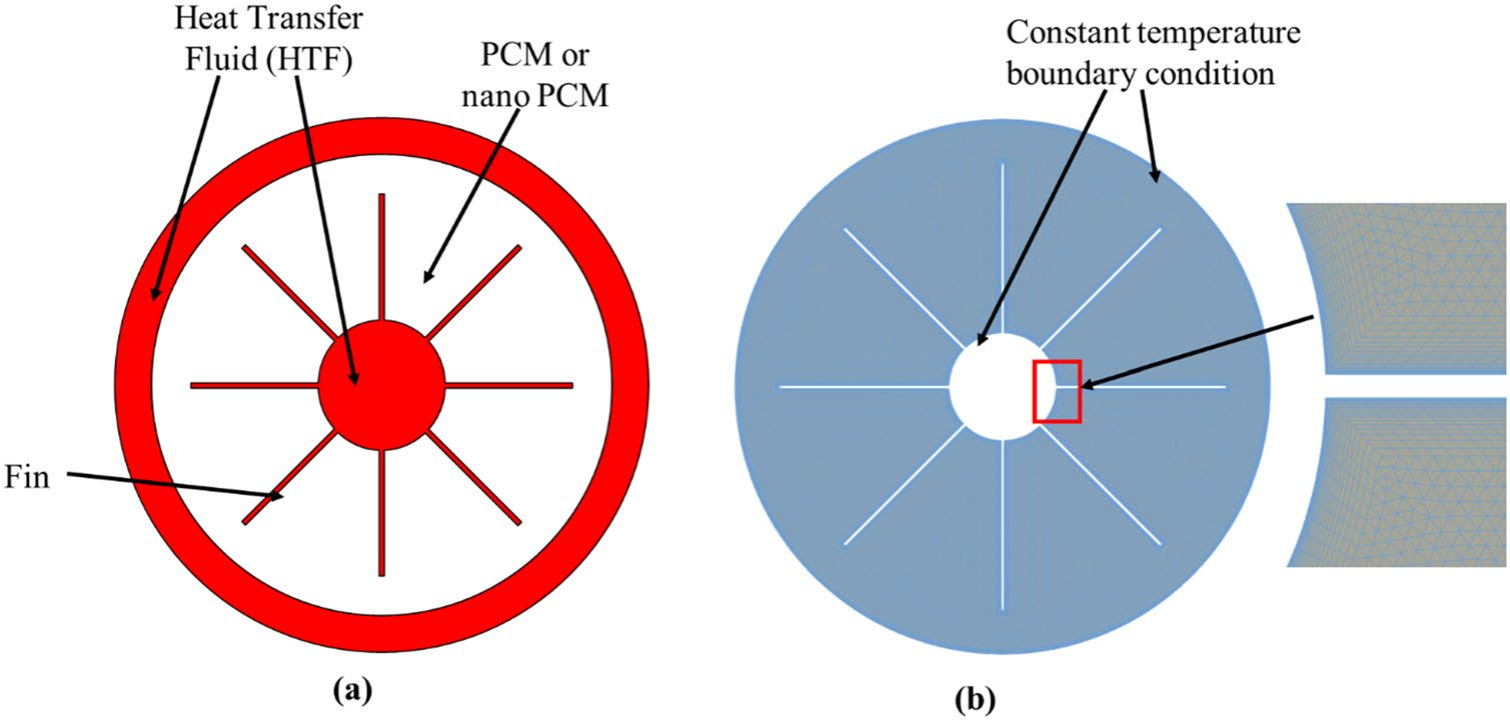
(**a**) Physical configuration of the triplex tube heat exchanger (TTHE) with internal fins, (**b**) simplified computational domain of the TTHE with internal fins.

For validation of the two-phase modeling of the NePCM, the experimental study^42^ of latent heat thermal storage system using a triplex tube heat exchanger (TTHE) is adopted. The physical configuration of the two-dimensional cross-section of TTHE is shown in Fig. 2a. This consist of two channels (inner and outer) in which heat transfer fluid (HTF) flows to control the inner and outer temperature boundary condition of the PCM. To enhance heat transfer, the configuration also includes 8 equally spaced fins attached to the inner tube. The outer tube, middle tube, and inner tube of the TTHE have diameters of 500 mm, 381 mm, and 76.2 mm, respectively while the fins are 121 mm long and 2 mm thick.

The PCM in the TTHE is made of paraffin RT82 while the nanoparticles are made of alumina (Al_2_O_3_). The thermophysical properties of pure PCM and pure alumina nanoparticles are shown in Table 4. A 10% NePCM was formed by uniformly mixing alumina nanoparticles with pure paraffin PCM in the liquid state. The nanoparticles have a diameter of 20 nm. The mixture (10% NePCM) was then allowed to solidify while keeping the nanoparticles uniformly distributed within the PCM. Hence, the thermophysical properties of the mixture can be computed from simple mixing empirical correlations using the properties of the constituent’s materials (pure PCM and nanoparticles) as earlier shown in Table 2. Note that NePCM simulations have used the same latent heat value due to the lack of a universal model (Table 4). While some studies have used a simple mixture model that reduces NePCM's latent heat linearly with increasing nanoparticle concentration, experimental investigations by Harikrishnan et al.^44^ and Sharma et al.^54^ have shown that such model does not match experimental measurements, and that a similar equation to the one employed for calculating the thermal properties of nanofluids cannot be straightforwardly used to predict the latent heat of NePCMs.

**Table 4:** Properties of PCM and alumina nanoparticles^42,53^.

Properties	Pure RT82 PCM	Alumina nanoparticles (Al_2_O_3_)
Specific heat capacity, ${C}_{p}$, J kg^−1^ K^−1^	2000	765
Thermal conductivity, $k$, W mK^−1^	0.2	36
Density, $\rho$, kg m^−3^	770	3600
Dynamic viscosity, $\mu$, kg ms^−1^	0.03499	–
Latent heat, L, J kg^−1^ K^−1^	176,000	–
Volumetric expansion coefficient, $\beta$, K^−1^	100 × 10^–6^	4.86 × 10^–6^
Solidus, ${T}_{s}$, K	350	–
Liquidus, ${T}_{l}$, K	358	–
Nanoparticle diameter, ${d}_{p}$, nm	–	20

**Figure 3:**
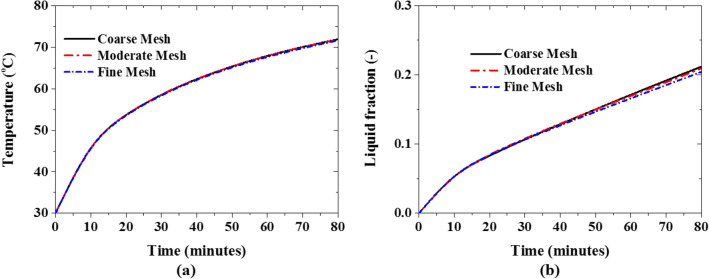
Mesh independent test: (**a**) temperature profile, and (**b**) liquid fraction profile.

The initial temperature of the solidified 10% NePCM is 30 °C and charging or melting of the NePCM is instigated by imposing a constant 90 °C temperature at the inner and outer walls of the computational domain (see Fig. 2b). At first, a mesh sensitivity study is performed using coarse mesh (60,000 control volumes), moderate mesh (120,000 control volumes), and fine mesh (220,000 control volumes). The transient average temperature (see Fig. 3a) of the PCM shows no significant dependence on mesh resolution while the PCM liquid fraction (see Fig. 3b) deviates slightly when a fine mesh is used. A moderate mesh resolution is, therefore, used henceforth.

**Figure 4:**
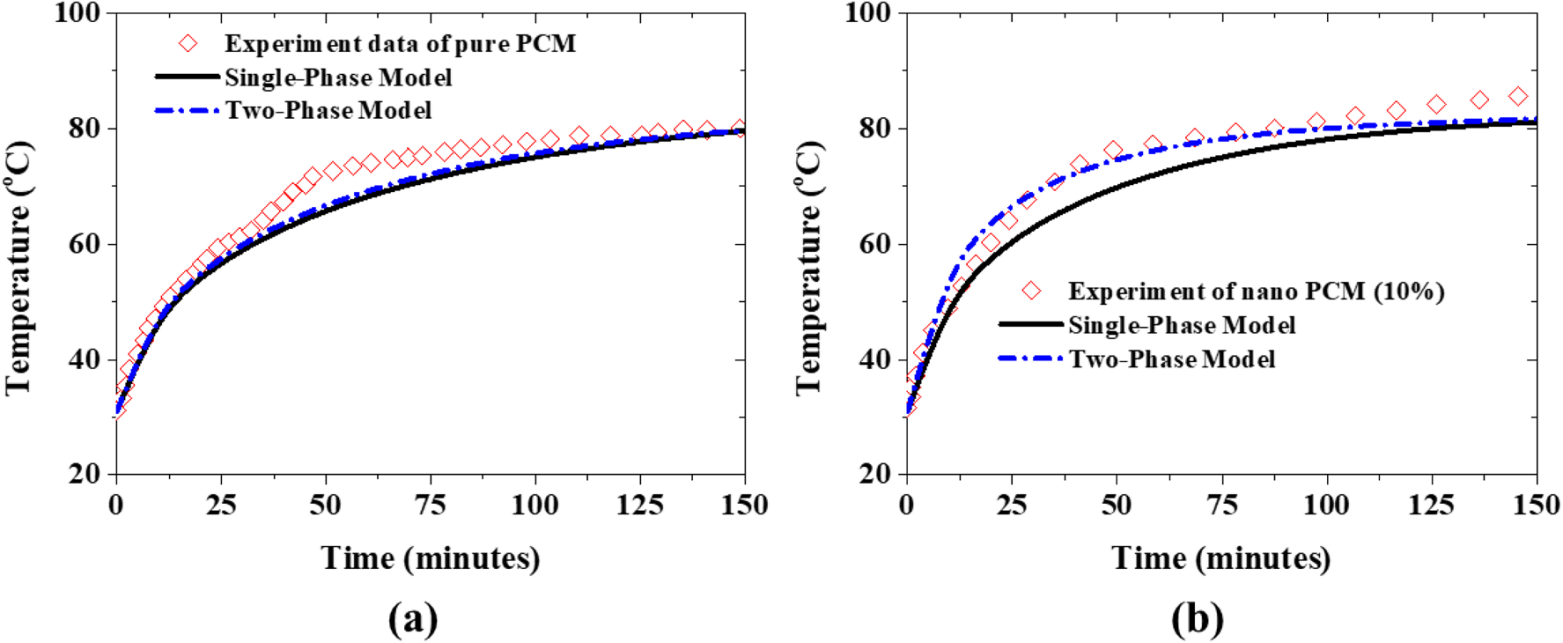
Comparison of the predicted temperature profile with experimental data: (**a**) pure PCM of 0% nanoparticles, and (**b**) nano PCM of 10% nanoparticles initial temperature = 30 °C.

**Figure 5:**
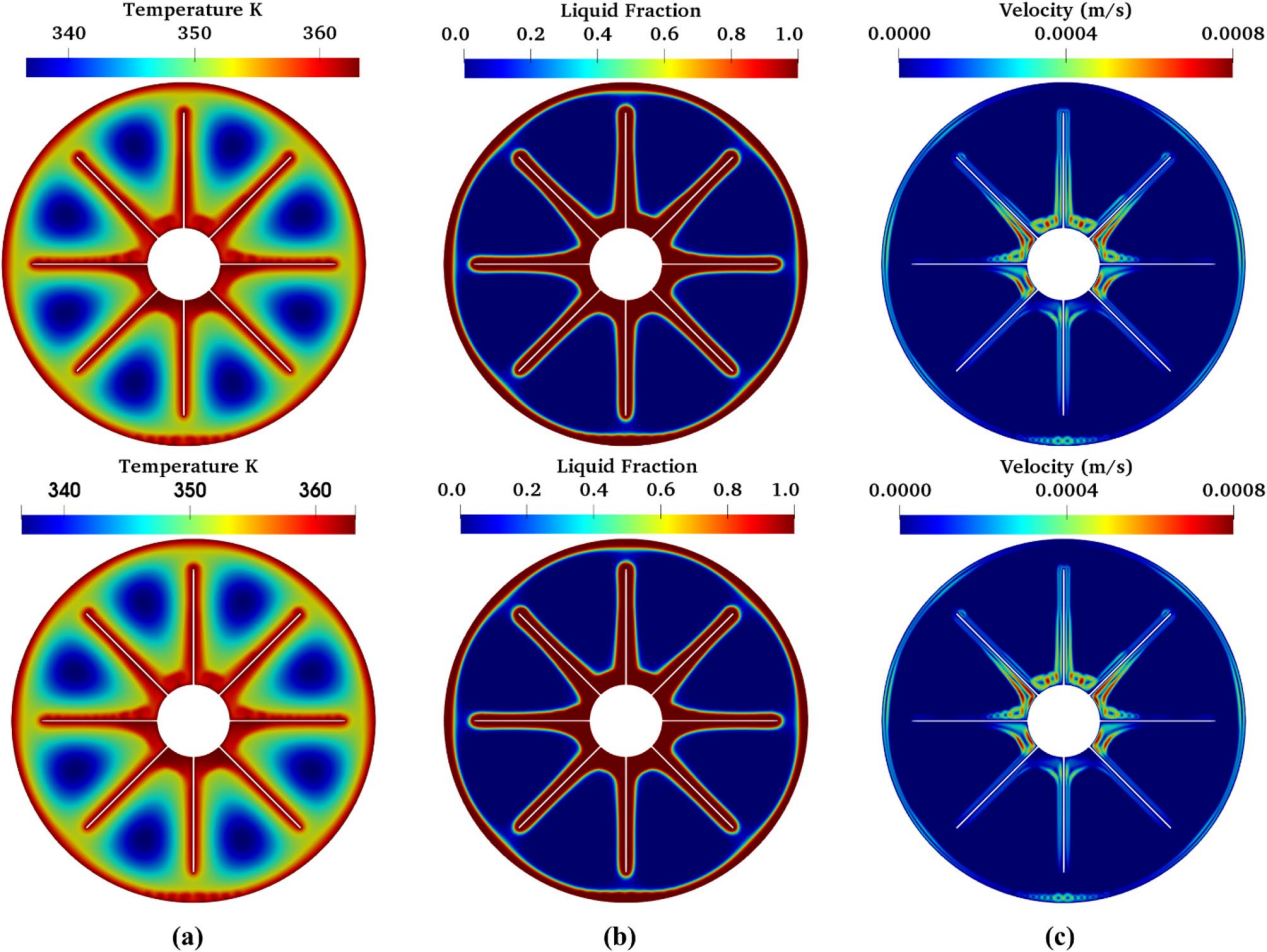
Predicted characteristics of pure PCM using single-phase model (top) and two-phase model (bottom): (**a**) temperature distribution, (**b**) liquid fraction distribution, and (**c**) velocity distribution at a simulation time of 120 min.

Moreover, both the single-phase model and two-phase model are used to simulate the charging process (melting) of both pure PCM (0% nanoparticles) and nano PCM (10% nanoparticles) to juxtapose their predictive accuracy vis-à-vis experimental data. For pure PCM, the experimental data of the average PCM transient temperature is predicted well by both single-phase and two-phase models as shown in Fig. 4a. Since the two-phase model reduces to a single-phase model at 0% nanoparticles concentration, the prediction by the two-phase model is then expectedly the same as that of the single-phase model (see Fig. 4a). This is further confirmed by the contours of temperature, liquid fraction, and velocity of the PCM which are qualitatively the same for both the two-phase model and single-phase model as can be seen in Fig. 5.

**Figure 6:**
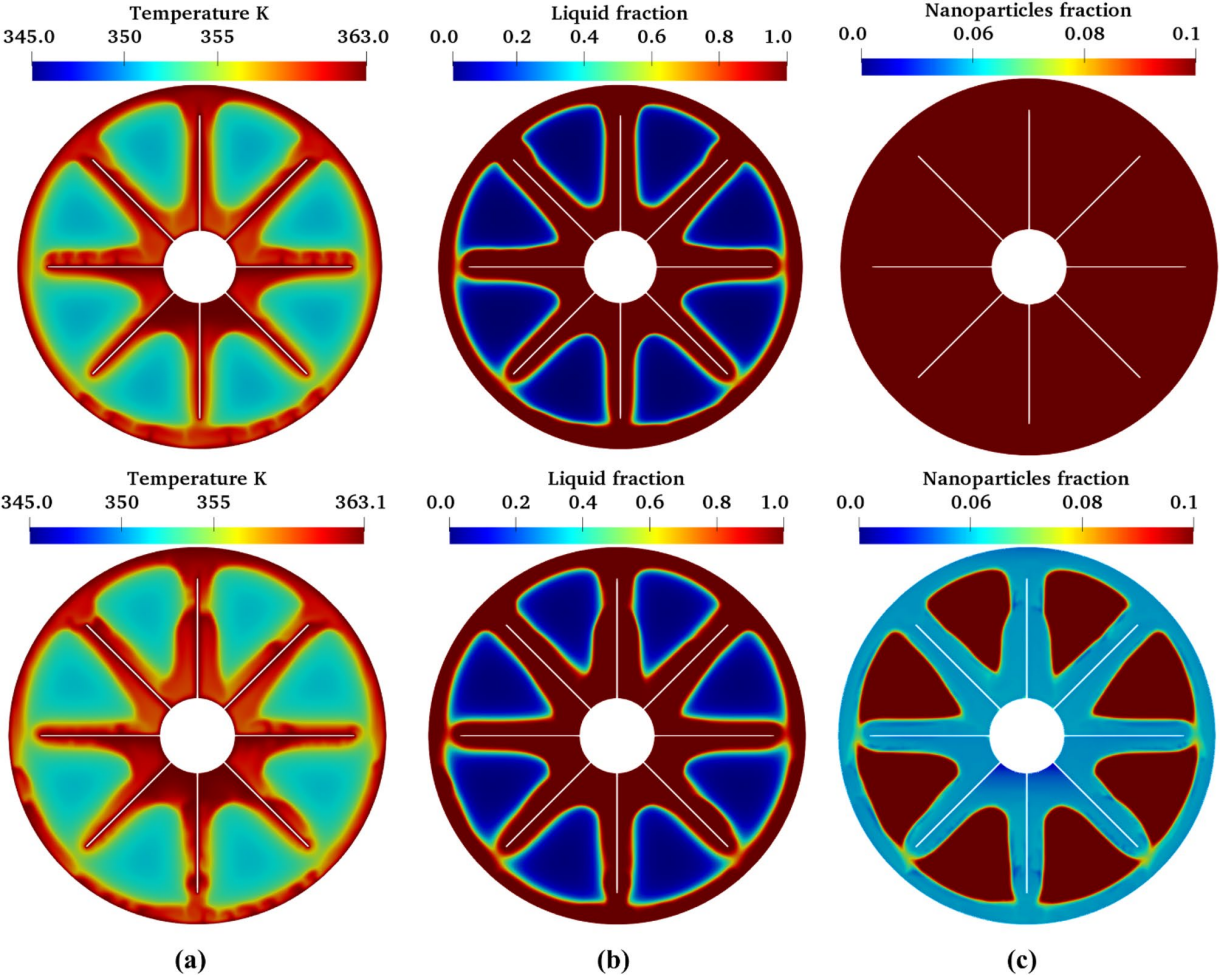
Predicted characteristics of 10% nano PCM using single-phase model (top) and two-phase model (bottom): (**a**) temperature distribution, (**b**) liquid fraction distribution, and (**c**) velocity distribution at a simulation time of 220 min.

However, for the 10% nanoparticles concentration test case, there is a distinct difference between the predicted average PCM transient temperature by the two-phase model and that by the single-phase model as shown in Fig. 4b. It is vital to stress here that the two-phase model produces much more accurate results than the single-phase model in this case. Thus, the migrations of nanoparticles within the PCM through sedimentation, Brownian diffusion, and thermophoresis are very essential for the accurate prediction of the thermal characteristics of a NePCM. The difference in the predictions also becomes accentuated by the contours of the temperature, liquid fraction, and nanoparticles concentration in the PCM which are clearly different (see Fig. 6). Interestingly, an expected non-uniform distribution of nanoparticles in the prediction by the two-phase model can be visualized in contrast to the uniform distribution of nanoparticles assumed by the single-phase model as can be seen in Fig. 6c. Therefore, it can be concluded that the two-phase model presented in this study can capture both the thermal characteristics of NePCM and the dynamic characteristics of the nanoparticles. This two-phase model is then used for the further investigation of the impact of nanoparticles migration on the performance of the NePCM over a couple of charging and discharging cycles as presented in the subsequent section of this article.

## Simulation of charging and discharging of the 0% and 10% nano PCM

The migration of nanoparticles through sedimentation, Brownian diffusion, and thermophoresis can cause a significant non-homogeneity of nanoparticle distribution within the base fluid which could have a substantial impact on the thermal performance of the NePCM in the long run. To investigate this phenomenon, three consecutive cycles of charging and discharging of 0% nano PCM and 10% nano PCM are performed using the two-phase approach. The charging process is accomplished by setting the temperature to 122 °C at the constant temperature boundaries (internal and external) of the geometry earlier described in Fig. 2. The charging (melting) process simulation continues until all the NePCM is fully melted. At this point (when the NePCM is fully melted), the temperature of the internal and external boundaries is changed to a constant of 30 °C to initiate the discharging (solidification) process and this continues until all the NePCM is completely solidified. This cycle is repeated for three times to investigate the long-term impact of the nanoparticles' migration on the thermal characteristics of the NePCM.

**Figure 7:**
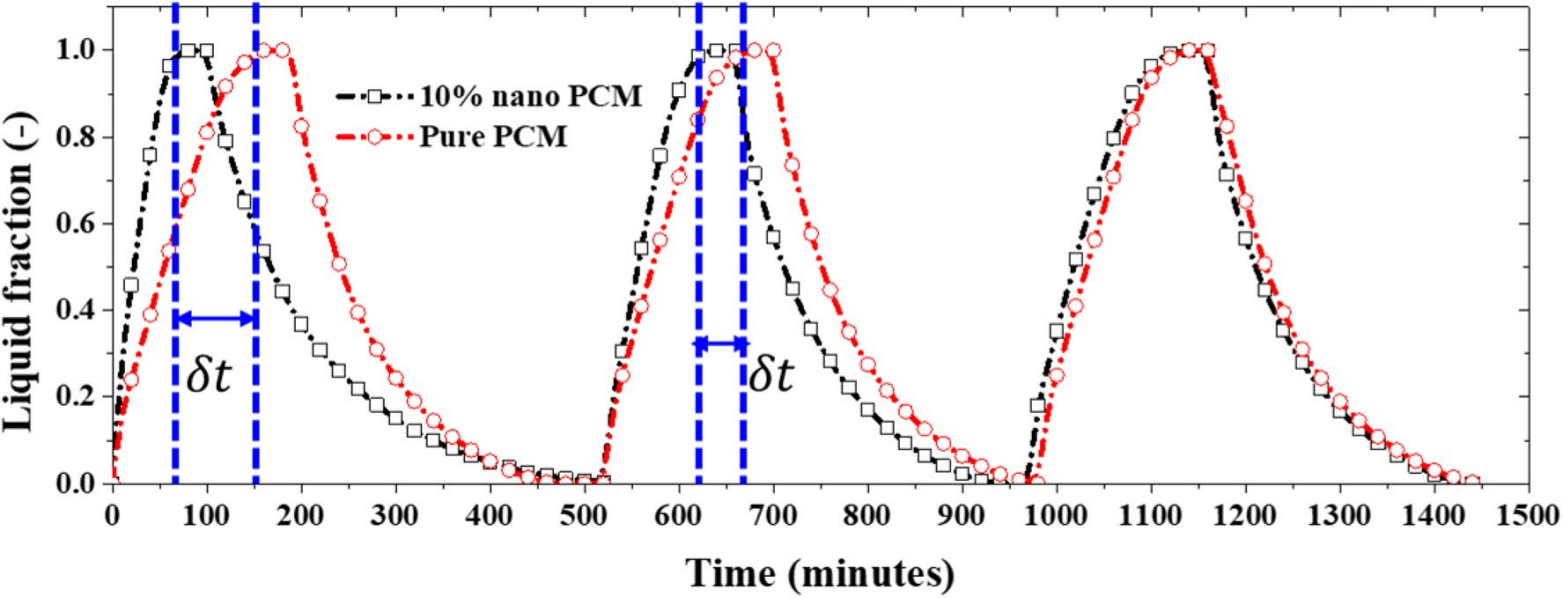
Charging and discharging of nano PCM at 10% nanoparticle volume fraction.

**Figure 8:**
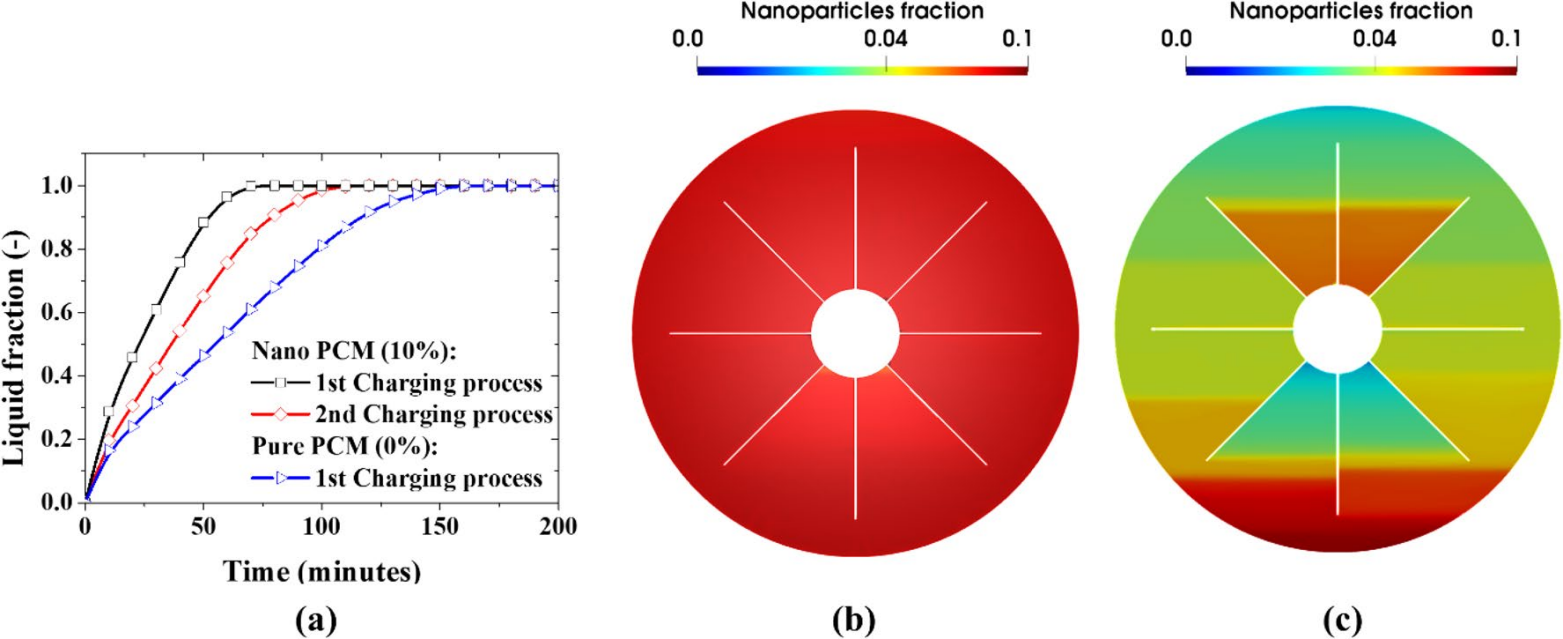
Comparison of two consecutive charging cycles: (**a**) plots of liquid fraction during the charging process, (**b**) nanoparticle fraction at the end of the 1st charging process, and (**c**) nanoparticle fraction at the end of the 2nd charging process.

During the first charging process, a significant improvement in the performance of the nano PCM is observed when 10% nanoparticles are mixed with the PCM as can be seen in Fig. 7. A fully melted condition is achieved for 10% NePCM at $\delta t=\sim 90$ min earlier than a 0% nano PCM (pure PCM) for the first charging process. However, a fully melted condition is observed for 10% NePCM at $\delta t=\sim 40$ min earlier than a pure PCM during the second charging process while $\delta t$ approaches zero for the third charging process. This shows about 50% degradation in the performance of the NePCM as shown in Fig. 8a. This is due to the continuous migration and deposition of nanoparticles within the PCM which causes substantial non-homogeneity in the concentration of nanoparticles as can be seen in Fig. 8b,c. At the end of the first charging process, the distribution of nanoparticles in the PCM is still relatively uniform (see Fig. 8b) but as the NePCM is continuously subjected to charging and discharging processes, the nanoparticles become concentrated in some regions within the NePCM due to the migration of the nanoparticles by sedimentation, Brownian diffusion, and thermophoresis (see Fig. 8c). In their experimental work, Ho et al.^53^ reported that for the case of a natural convection flow in an enclosure filled with nanofluid at low nanoparticles concentration (less than 1%) the convective heat transfer coefficient is enhanced in comparison to the base fluid, while for higher nanoparticles concentration a trend of heat transfer deterioration in comparison to the base fluid was reported. Similarly, during the melting/solidification processes of the PCM, the liquid phase is in direct contact with hot and cold surfaces, hence, a natural convection flow occurs (see for example Fig. 1 in the manuscript). Accordingly, the heat transfer impairment as a function of nanoparticles concentration, reported in Ho et al.^53^, also applies during the melting/solidification processes. Notably, this impairment does not seem to impact the resulting transient temperature profile illustrated in Fig. 4 for the first cycle, but it becomes more significant in the following cycles.

Most investigators normally use one charging process or one discharging process to evaluate the enhancement of performance by nanoparticles in PCM. This approach does not give the complete picture because it might become practically impossible to keep the nanoparticles distribution uniform over a long operation time of the NePCM (many continuous cycles of charging and discharging). As demonstrated in this study, the performance of the NePCM tends to deteriorate over a long operation time involving many charging and discharging progress because of the non-uniformity of nanoparticles concentration caused by migration and deposition of nanoparticles in the PCM.

## Conclusion

A two-phase modeling approach has been demonstrated in this study for the prediction of the thermal characteristics of the nanoparticles-enhanced phase change material (NePCM). The two-phase model captures the slip motions of nanoparticles in the PCM due to sedimentation, Brownian diffusion, and thermophoresis. Previous investigations of nano PCM usually adopted a single-phase modeling approach which becomes inadequate when the NePCM is operated over several cycles of charging and discharging as evident in this study.

In cases where the nano PCM is operated in a single charging or discharging process with an initial condition of homogeneous distribution of nanoparticles, a substantial enhancement of the heat transfer performance of the nano PCM is observed relative to pure PCM. Expectedly, this agrees with the findings in previous studies where different percentages of enhancement have been reported. However, the continuous operation of the nano PCM over a couple of charging and discharging processes has shown a significant deterioration of NePCM heat transfer performance. This is due to the non-homogeneity of nanoparticle concentrations caused by their migrations due to; sedimentation, Brownian diffusion, and thermophoresis which results in nanoparticle deposition. This degradation of the heat transfer performance of NePCM, at high nanoparticles concentration (> 1%), is brought to bear by the two-phase model in which relevant nanoparticle migration mechanisms are accounted for. A comparison of the first charging and second charging of NePCM with the first charging of pure PCM shows that the performance of the NePCM at the second charging is reduced significantly by about 50% albeit still better than the pure PCM case by about 20%. Therefore, it can be concluded that the investigation of NePCM using a single-phase model over a single cycle of charging or discharging does not provide a complete story concerning the heat transfer performance of the NePCM. Instead, a two-phase model which duly accounts for the relevant nanoparticles slip mechanism is recommended for the holistic investigation of the thermal characteristics of NePCM operated over several charging and discharging processes.

## Data Availability

The datasets generated during the current study are available from the corresponding author on reasonable request.

## List of symbols

$A$: Settling or sedimentation constant
${C}_{pp}$: Specific heat capacity of the nanoparticles (J kg^−^^1^ K^−^^1^)
${C}_{pm}$: Specific heat capacity of mixture (PCM and nanoparticle) (J kg^−^^1^ K^−^^1^)
${C}_{pbf}$: Specific heat capacity of the base fluid (PCM) (J kg^−^^1^ K^−^^1^)
${D}_{T}$: Thermophoretic diffusion coefficient (m^2^ s^−^^1^)
${D}_{B}$: Brownian diffusion coefficient (m^2^ s^−^^1^)
$${d}_{p}$$: Nanoparticles diameter (m)
$${g}_{k}$$: Buoyancy term
${k}_{np}$: Thermal conductivity of the nanoparticles (W m^−^^1^ K^−^^1^)
${k}_{m}$: Thermal conductivity of mixture (PCM and nanoparticle) (W m^−^^1^ K^−^^1^)
${k}_{bf}$: Thermal conductivity of the base fluid (PCM) (W m^−^^1^ K^−^^1^)
${k}_{B}$: Boltzmann constant (m^2^ kg s^−^^2^ K^−^^1^)
$L$: Latent heat (J kg^−^^1^ K^−^^1^)
${S}_{m}$: Momentum source term
${S}_{pm}$: Nanoparticle transport source term
$${S}_{T}$$: Thermophoretic constant
${S}_{h}$: Enthalpy source term
$T$: Temperature of the single fluid (single-phase model) (K)
$${T}_{s}$$: Solidus temperature (K)
$${T}_{me}$$: Melting temperature ($$=({T}_{s}+{T}_{l})/2$$) (K)
$${T}_{l}$$: Liquidus temperature (K)
$${T}_{Ref}$$: Reference temperature (K)
$u$: Single fluid velocity vector (m s^−^^1^)
${u}_{pm}$: Relative velocity of nanoparticles due to sedimentation (m s^−^^1^)
${u}_{m}$: Mixture velocity vector (two-phase model) (m s^−^^1^)
${u}_{T}$: Relative velocity of nanoparticles due to thermophoresis diffusion (m s^−^^1^)
${u}_{S}$: Settling termina velocity vector (m s^−^^1^)
${u}_{B}$: Relative velocity of nanoparticles due to Brownian diffusion (m s^−^^1^)
${\beta }_{p}$: Volumetric expansion coefficient of nanoparticles (K^−^^1^)
${\beta }_{m}$: Volumetric expansion coefficient of mixture (PCM and nanoparticle) (K^−1^)
${\beta }_{bf}$: Volumetric expansion coefficient of base fluid (PCM) (K^−1^)
${\mu }_{m}$: Dynamic viscosity of the mixture (PCM and nanoparticle) (kg m^−^^1^ s^−^^1^)
${\mu }_{bf}$: Dynamic viscosity of the base fluid (PCM) (kg m^−^^1^ s^−^^1^)
${\rho }_{p}$: Density of the nanoparticles (kg m^−^^3^)
${\rho }_{m}$: Density of the mixture (PCM and nanoparticle) (kg m^−^^3^)
${\rho }_{bf}$: Density of the base fluid (PCM) (kg m^−^^3^)
$\varphi$: Volumetric concentration of nanoparticles (fractional)
